# Fibre-optic metadevice for all-optical signal modulation based on coherent absorption

**DOI:** 10.1038/s41467-017-02434-y

**Published:** 2018-01-12

**Authors:** Angelos Xomalis, Iosif Demirtzioglou, Eric Plum, Yongmin Jung, Venkatram Nalla, Cosimo Lacava, Kevin F. MacDonald, Periklis Petropoulos, David J. Richardson, Nikolay I. Zheludev

**Affiliations:** 1 Optoelectronics Research Centre, University of Southampton, Southampton, SO17 1BJ UK; 2 Centre for Photonic Metamaterials, University of Southampton, Southampton, SO17 1BJ UK; 3 Centre for Disruptive Photonic Technologies, School of Physical and Mathematical Sciences and The Photonics Institute, Nanyang Technological University, Singapore, 637371 Singapore

## Abstract

Recently, coherent control of the optical response of thin films in standing waves has attracted considerable attention, ranging from applications in excitation-selective spectroscopy and nonlinear optics to all-optical image processing. Here, we show that integration of metamaterial and optical fibre technologies allows the use of coherently controlled absorption in a fully fiberized and packaged switching metadevice. With this metadevice, which controls light with light in a nanoscale plasmonic metamaterial film on an optical fibre tip, we provide proof-of-principle demonstrations of logical functions XOR, NOT and AND that are performed within a coherent fibre network at wavelengths between 1530 and 1565 nm. The metadevice has been tested at up to 40 gigabits per second and sub-milliwatt power levels. Since coherent absorption can operate at the single-photon level and with 100 THz bandwidth, we argue that the demonstrated all-optical switch concept has potential applications in coherent and quantum information networks.

## Abstract (web summary)

Here, the authors show that integration of metamaterial and optical fibre technologies enables all-optical XOR, NOT and AND logical functions that are performed at up to 40 gigabits per second with few femtojoules per bit energy consumption within a coherent fully fiberized network.

## Introduction

All-optical signal processing fundamentally relies on modulation of one optical signal with another. Therefore all-optical logical functions have long been perceived as the exclusive domain of nonlinear optics^1^, which requires a minimum level of intensity to activate the nonlinear material response and faces trade-offs between magnitude and speed of the nonlinearity involved^2–6^. However, recently it was shown that an effective nonlinear response may be derived from coherent interaction of light with light on linear materials of substantially sub-wavelength thickness^7^. In contrast to conventional optical nonlinearities, the effect has been shown to allow intensity-independent control over absorption of light, from almost 0% to almost 100%^8^, with 100 THz bandwidth^9,10^ and even for single-photon signals^11^. The concept has enabled all-optical control of luminescence^12,13^, redirection of light^14,15^ as well as control of nonlinear^16^, polarization^17^ and quantum^18,19^ effects in films of nanoscale thickness and excitation-selective spectroscopy^20^. In particular, it has been predicted that coherent interaction of light waves on lossy ultrathin films could perform signal processing functions^7^ and proof-of-concept experiments in the static regime have been reported^21,22^.

Here, we report the realization of a switching network using a fully packaged, fiberized metamaterial device based on this concept. It uses a plasmonic nanostructure of substantially sub-wavelength thickness as a switchable absorber that allows the absorption of one optical pulse to be controlled by another coherent optical pulse. We demonstrate nonlinear input–output characteristics and all-optical operations analogous to logical functions NOT, AND and XOR at both kHz and GHz bitrates, in a fiberized configuration assembled from standard telecoms components, at wavelengths between 1530 and 1565 nm.

## Results

### Coherent metamaterial absorption in a fibre network

**Fig. 1:**
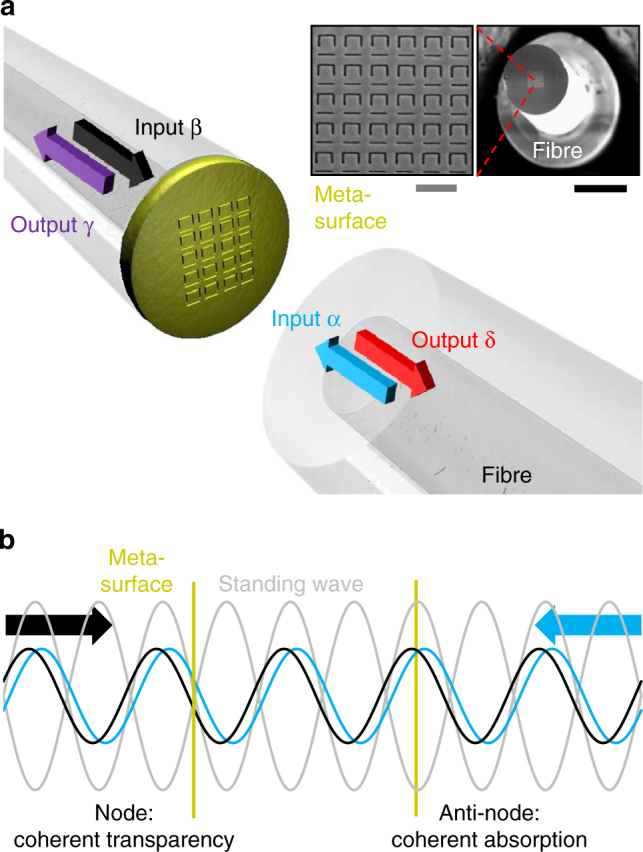
Coherent interaction of light with light on a metasurface. **a** Coherent optical input signals α and β interact on a metasurface absorber, generating output signals γ and δ. The metasurface has been fabricated by nanostructuring the central 25 × 25 μm^2^ of a 70-nm-thick gold layer covering the cleaved end-face of a polarization-maintaining single-mode silica fibre (inset scanning electron microscope images, black scale bar 100 μm, grey scale bar 1 μm). **b** The counterpropagating coherent input signals form a standing wave wherein the metasurface can be located at a position of destructive interference of electric fields (node) where absorption is suppressed or at a position of constructive interference (anti-node) where absorption is increased. In the ideal case, absorption can correspondingly be controlled from 0 to 100%

As illustrated in Fig. 1a, the functionality of the network is based on controlling absorption of light with light on an ultrathin metamaterial absorber. It has two bidirectional ports, i.e. two inputs, α and β, and two outputs, γ and δ. The input waves propagate in opposite directions and, provided that they are mutually coherent, co-polarized and of equal intensity, they will form a standing wave with electric field nodes and anti-nodes (Fig. 1b). A sufficiently thin film may thus be placed at a node, where the electric field is zero due to destructive interference, or at an anti-node where the electric field amplitude is enhanced by constructive interference. Since truly planar structures interact with normally incident waves only via the electric field^23^, this implies that a planar thin film placed at a node will be perfectly transparent, while the same thin film will be strongly excited if placed at an anti-node. With respect to absorption, which is limited to 50% in planar materials illuminated by a travelling plane wave^24^, standing wave illumination allows absorption to be controlled from 0 to 100% in the ideal case^7^. Such performance in the optical part of the spectrum, as is relevant to optical fibre technology, may be approximated with materials that are thin compared to the optical wavelength and exhibit equal levels of transmission and reflection in addition to around 50% travelling wave absorption. This combination of parameters may be achieved for example in nanostructured plasmonic metamaterials^8^ and 30-layer graphene^11,25^.

**Fig. 2:**
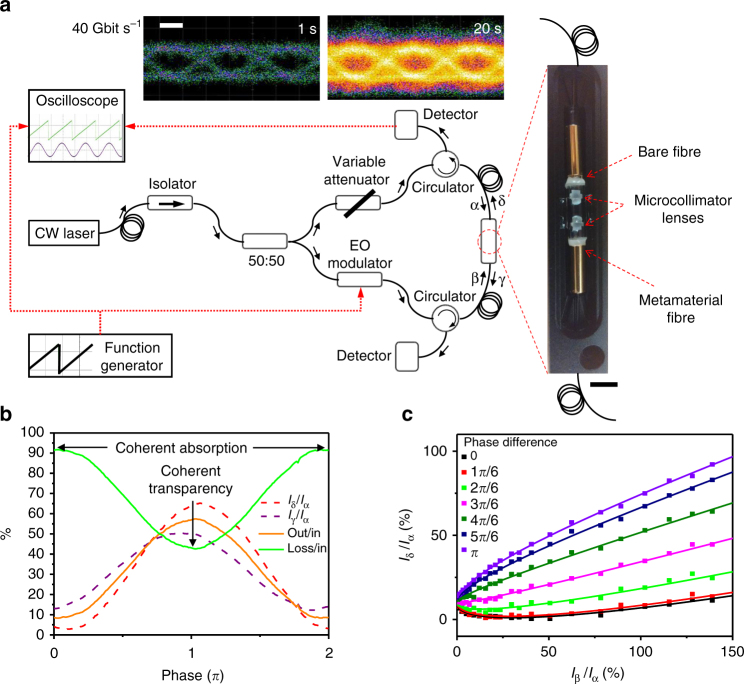
The packaged metadevice and its properties. **a** Schematic representation of the fully fiberized experimental setup with a photograph of the packaged metadevice (without lid, black scale bar 5 mm) consisting of the metasurface-covered fibre (Fig. 1a) coupled to a bare fibre end using a pair of microcollimator lenses. The inset shows eye diagrams of the intensity of output channel δ recorded for intensity modulation of input channel β at 40 Gbit s^−1^, where colour indicates counts and the white scale bar indicates 10 ps. **b** Measured output intensities *I*_γ_ and *I*_δ_ (relative to *I*_α_) as well as the total output power and metadevice losses (relative to the total input power) as a function of the phase difference between the input signals at the metasurface at a wavelength of 1550 nm. **c** Measured output intensity *I*_δ_ (data points) relative to the fixed input intensity *I*_α_ as a function of input intensity *I*_β_ for various phase differences between the input beams, with fits (lines), again at 1550 nm

The planar absorber used here is a plasmonic metamaterial consisting of a 70-nm-thick gold film perforated with an array of asymmetrically split ring apertures, as previously deployed in free-space demonstrations of coherent light absorption and transparency^8^. The dependence of this structure’s transmission, reflection and absorption characteristics on aperture size and geometry^26^ is well understood, allowing for easy optimization throughout the optical telecommunications bands. Our switch operates at wavelengths around *λ* = 1550 nm, where its 70 nm thickness corresponds to *λ*/22. The metamaterial structure was fabricated by thermal evaporation of gold and subsequent focused ion beam milling on a 25 × 25 μm^2^ area covering the core of a cleaved polarization-maintaining single-mode silica fibre (see inset to Fig. 1a), with the symmetry axis of the metamaterial aligned to the slow axis of the fibre (see Methods section for details). The fibre output was coupled to a second cleaved polarization-maintaining optical fibre using two microcollimator lenses to realize an in-line fibre metadevice (Fig. 2a).

The metadevice is terminated with standard FC/APC fibre connectors and it was characterized in a fibre interferometer assembled from standard polarization-maintaining fibre components (Fig. 2a). The output of a fibre-coupled CW laser was split along two paths of similar length, with one path containing an electro-optical phase or intensity modulator and the other containing a variable attenuator to allow balancing of the power propagating along the two paths. The paths were then recombined within the metadevice and the output signals were detected via circulators using an oscilloscope (see Methods section for a more detailed description). We note that practical applications would require some means of active stabilization of the optical path lengths, however the system shown here is sufficient to characterize the principle of operation of the fiberized switch. This is illustrated by the eye diagrams in Fig. 2a, which show that the eye closes on a timescale of seconds due to phase drift in the interferometer.

Figure 2b shows the phase-dependent output intensities *I*_γ_ and *I*_δ_ relative to the input intensity *I*_α_ = *I*_β_ as a function of the phase difference between the inputs at a wavelength of 1550 nm. The overall output power can be controlled from about 9% to about 57% of the total input power, where a low output level corresponds to constructive interference of the incident waves on the metasurface and thus coherent absorption, while a high output level corresponds to coherent transparency. Both output signals display a similar phase-dependence; however, the phase-dependent output intensity *I*_δ_ offers somewhat higher contrast and therefore we focus on this output. It should be noted that, for an ideal metadevice containing a perfectly symmetric ultrathin absorber showing 50% single-beam absorption (with no other loss mechanisms) and equal reflection and transmission for both directions of propagation, both output intensities would be identical and modulated from complete absorption to perfect transmission. Differences between the measured output channels arise in the present case from the asymmetric construction of our metadevice that contains a metasurface fabricated on the glass/air interface of one of the optical fibres. The whole metadevice exhibits about 24% single-beam transmission, 18% (8%) reflection and 58% (68%) losses for a single input signal α (β). However, only part of these losses correspond to metasurface absorption that can be coherently controlled, while other sources of loss include the fibre connections of the metadevice, scattering and unwanted reflections within the microcollimator and fabrication imperfections such as imperfect alignment of the metasurface orientation with the slow axis of the fibres.

The nonlinear functionality of the switch is illustrated by Fig. 2c, which shows how the output intensity *I*_δ_ depends on the input intensity *I*_β_ while *I*_α_ remains constant for various phase differences between the input beams. The measured output intensity *I*_δ_ as a function of input *I*_β_ is nonlinear and generally follows the behaviour predicted by Fang et al.^7^. For input phase differences of less than π/2 it is also nonmonotonic—counterintuitively the output intensity decreases with increasing input intensity and reaches a minimum before increasing when *I*_β_ becomes large. For an input phase difference of π/2, changes in the measured output intensity *I*_δ_ are approximately proportional to changes in the input intensity *I*_β_. For larger input phase differences, *I*_δ_ flattens with increasing *I*_β_, but is steep for small *I*_β_ suggesting possible applications in small signal amplification. Thus, the results presented in Fig. 2b, c show that large changes of the metadevice output result from modulation of phase or intensity of one of the metadevice inputs.

### All-optical signal processing

**Fig. 3:**
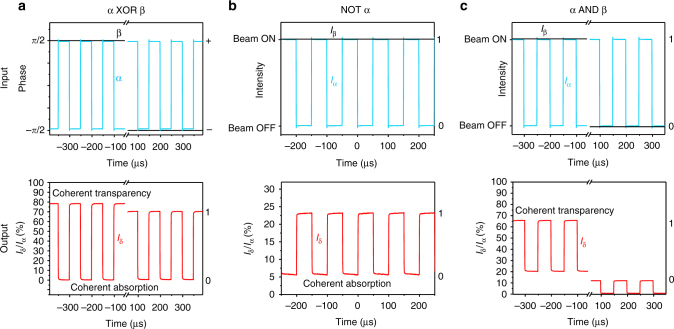
All-optical signal processing at 10 kHz at a wavelength of 1550 nm. **a** XOR function between phase-modulated input signals α and β producing an intensity-modulated output based on coherent absorption of identical bits and coherent transparency for opposing bits. **b** NOT function on a single intensity-modulated signal α. The inversion of signal α in the presence of beam β (which is always on) results from coherent absorption of incoming signal pulses when the metasurface is located at a standing wave anti-node. **c** AND function between intensity-modulated signals α and β resulting from coherent transparency of the metasurface for simultaneous illumination from both sides when the metasurface is located at a standing wave node. The logical states are indicated on the right-hand side of each graph. Minor signal distortions are due to the limited bandwidth of the waveform generator

All-optical signal processing operations with input/output relations analogous to logical functions may now be realized in the network by exploiting coherent transparency and/or coherent absorption in the metadevice. In what follows, binary logical states encoded in beams of equal intensity but opposite phase in the metasurface plane are denoted '+' and '−', while opposing states encoded as low/high intensity are denoted '0'/'1'. Consider, in the first instance, the case of mutually coherent, binary, phase-modulated input signals + and −: constructive interference of identical bits α and β will lead to coherent absorption on the metasurface, while destructive interference of opposing bits will lead to coherent transparency, producing an intensity-modulated (0/1) output α XOR β. The behaviour of an ideal switch is summarized in Table 1 and the measured behaviour of the experimental metadevice is shown in Fig. 3a, for a modulation frequency of 10 kHz. The switch clearly presents XOR functionality with high contrast (>10×) between the output states. The intensity of the output logical 1 is about 30% lower than in the ideal case due to losses within the metadevice. Note that the XOR function can be inverted to α XNOR β by providing an additional external phase-shift *θ*_ext_ = π to one of the input signals. Furthermore, a fixed input signal β of + or − could be used to map the phase-modulated signal α to an intensity-modulated signal with (NOT α) or without (IDENTITY α) inversion.

**Table 1:** Logical functions between mutually coherent, equal intensity, phase-modulated input bits α and β (*I*_α_ = *I*_β_ = 1)

Input phase states		Ideal output intensities *I*_γ_ = *I*_δ_	
		*θ*_ext_ = 0	*θ*_ext_ = π
α	β	α XOR β	α XNOR β
+	+	0	1
+	−	1	0
−	+	1	0
−	−	0	1

The network can also perform signal processing operations analogous to logical functions on intensity-modulated input data. The simplest example is a NOT function. Such inversion of an intensity-modulated signal α is achieved by leaving input beam β always on with its phase adjusted such that coherent absorption will occur for simultaneous illumination of the metasurface from both sides (*θ*_ext_ = 0). Input pulses α (logical 1) will be coherently absorbed resulting in low output (logical 0). On the other hand, low input signals α (logical 0) will allow light from input β to reach the outputs (logical 1). For an ideal metadevice, the expected output intensities are 0 and 25% of the input intensity, respectively; our experimental device achieves about 5 and 23% at a modulation frequency of 10 kHz, which is more than sufficient to distinguish the logical states (Fig. 3b).

While the NOT function was based on coherent absorption, an AND function between binary intensity-modulated signals can be realized by exploiting coherent transparency. In this case, a phase shift is applied to one input signal, such that simultaneous illumination of the metasurface from both sides leads to coherent transparency (*θ*_ext_ = π). For an ideal device, this would lead to 100% output intensity for interaction of two pulses on the metasurface and at least 4× lower output intensity for any other combination of input bits (Table 2). Experimentally we observe the AND function with more than 3× contrast between the logical output states at a modulation frequency of 10 kHz. In principle, other logical functions including XOR and OR for intensity-modulated signals can be realized for suitable choices of *θ*_ext_^22^.

**Table 2:** Logical function α AND β between mutually coherent, intensity-modulated input bits α and β

Input states		Ideal output intensities *I*_γ_ = *I*_δ_
		*θ*_ext_ = π
α = *I*_α_	β = *I*_β_	α AND β
1	1	1
1	0	0.25
0	1	0.25
0	0	0

### Gigabits per second and beyond

**Fig. 4:**
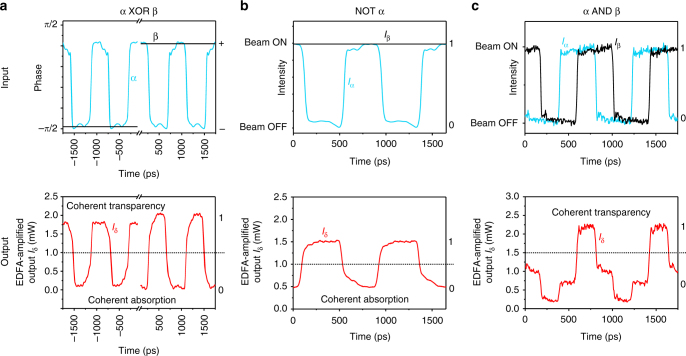
All-optical signal processing at 1.2 GHz at a wavelength of 1550 nm. **a** XOR function between phase-modulated input signals α and β producing an intensity-modulated output based on coherent absorption of identical bits and coherent transparency for opposing bits. **b** NOT function on a single intensity-modulated signal α in the presence of a constant beam β, resulting from coherent absorption of incoming signal pulses when the metasurface is located at a standing wave anti-node. **c** AND function on two intensity-modulated signals α and β resulting from coherent transparency of the metasurface for simultaneous illumination from both sides when the metasurface is located at a standing wave node. The elevated noise level in **c**, as compared to **a**, **b**, is due to a change in the experimental configuration (described in Methods). The logical states are indicated on the right-hand side of each graph

**Fig. 5:**
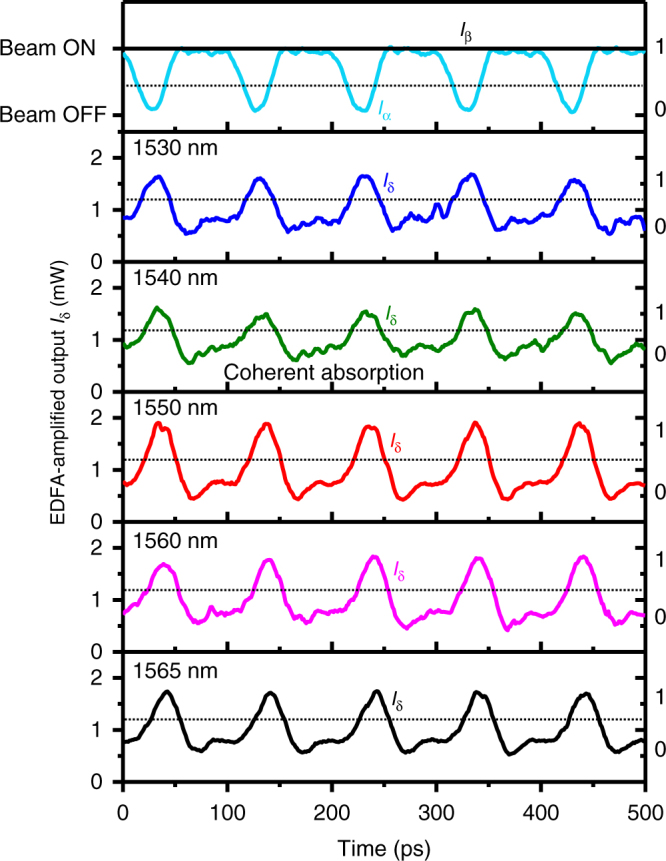
Broadband inversion NOT α of a 40 Gbit s^−1^ input signal α at wavelengths from 1530 to 1565 nm. The input signal corresponds to an intensity-modulated bit pattern 1011 repeating at 10 GHz (top); corresponding output traces for different wavelengths (below) show that the metadevice inverts the bit pattern in all cases. Beam β is continuously in the on state (logical 1). The logical states 1 and 0 are indicated on the right-hand side and separated by a horizontal dotted line on each graph

In practical systems, optical signals transmitted by optical fibres are modulated at GHz frequencies rather than kHz, and also make use of a range of optical wavelengths. We therefore tested the metadevice at modulation frequencies 5–6 orders of magnitude higher than presented in Fig. 3 and at wavelengths ranging from 1530 to 1565 nm (telecommunications C-band), as illustrated in Figs. 4 and 5. To this end, the output of the fibre interferometer was amplified with an erbium-doped fibre amplifier (EDFA) which provided an average output power of 1 mW. This conveniently ensures that the threshold power between logical 0 and logical 1 will always be close to 1 mW. Figure 4a shows the XOR function on phase-modulated signals (as described above) now at a modulation frequency of 1.2 GHz, while Fig. 4b shows the NOT function on an intensity-modulated signal also at 1.2 GHz. An AND function was realized by intensity modulation of the laser light entering the interferometer (i.e. modulation before the 50:50 splitter shown in Fig. 2a) and the introduction of a path difference in the interferometer arms to delay the modulated signals α and β relative to one another as illustrated by Fig. 4c. This is equivalent to an intensity-modulated bit sequence 0011 in channel α and 1001 in channel β, resulting in an output 0001, i.e. α AND β, in the detected output channel.

Figure 5 shows the NOT function of the switching network at a frequency of 10 GHz. The periodic input signal α was generated with a bit pattern generator and is equivalent to a bit sequence of 1011, which is inverted to become 0100 at a rate of 40 Gbit s^−1^. Measurements at wavelengths from 1530 to 1565 nm show successful signal inversion, thereby illustrating the broadband nature of the underlying coherent absorption effect across the full wavelength range of the tuneable laser used in the experiment. With 50 μW peak power in each input channel and a 25 ps pulse duration per bit, the modulator’s energy consumption is 2.5 fJ per bit, which corresponds to about 20,000 photons per bit. Given that coherent absorption of single photons has been demonstrated^11^, we expect that energy consumption in the attojoule per bit regime should be possible with a sufficiently sensitive detection system.

The modulation results obtained at GHz frequencies are similar to those obtained at kHz frequencies. At GHz frequencies, some distortion is apparent in both the signals used to drive the phase and intensity modulators as well as the measured output signal, where contrast is slightly reduced. These distortions arise from the frequency response and background noise of the modulators and amplifiers used in the experiments. While our experimental equipment does not allow us to test the performance of the modulator in the fibre environment beyond 40 Gbit s^−1^, we expect that the switching network with metamaterial absorber can in principle operate at much higher frequencies. Indeed, the underlying phenomena of coherent absorption and coherent transparency in plasmonic metamaterials occur on timescales as short as 10 fs, implying a potential bandwidth on the order of 100 THz^10^. Based upon the spectral width of its plasmonic absorption peak, the metamaterial used in the present study may be expected to efficiently absorb pulses as short as 40 fs, corresponding to a potential bandwidth of tens of THz (Supplementary Note 1). However, such bandwidth will be difficult to realize in a fiberized switch due to dispersion limitations of the fibres.

## Discussion

Even though we report a proof-of-principle demonstration, our results show that the coherent interaction of light with light on ultrathin films can be used to perform signal processing functions with high bandwidth and high contrast on signals carried by telecoms fibres. Improved device performance would result from a more symmetric design wherein the nanoscale thickness absorber is in contact with the same material on both sides, which may be achieved for instance by depositing a thin glass layer over the metasurface or via a bespoke splicing technique. The metamaterial design itself could also be improved to achieve 50% single-beam absorption and identical 25% transmission and reflection characteristics from both sides as well as polarization independence. Another option may be to replace the metamaterial with multi-layer graphene^11,25^.

All-optical signal processing applications based on coherently controlling the absorption of light with light promise extremely high bandwidth and extremely low energy requirements, but they will require mutually coherent signals and phase stability. Mutual coherence is most easily achieved in local systems, where multiple signals are derived from the same seed laser. Indeed, as locally coherent networks become part of the mainstream telecommunications agenda^27–29^, coherent all-optical data processing may become a realistic proposition, particularly in miniaturized integrated optics and silicon photonic chips^30^, where phase stability is more easily achieved than in large-scale fibre networks. In order to go beyond simple single-step logical functions, cascading of multiple coherent operations will also need to be explored. Such cascading is likely to require signal regeneration techniques as the XOR function converts phase-shift keying to amplitude-shift keying (though in principle without insertion loss and with unlimited contrast), while the AND function suffers from limited contrast of 6 dB (though in principle without insertion loss) and the NOT function suffers from insertion loss of 6 dB (in principle with unlimited contrast).

It is notable that metasurfaces can be engineered to enable a broad range of switching and control functions, which may be used to implement various signal processing functionalities^7,22,31^. Devices based on coherent perfect absorption (as considered here) and lossless devices are limiting cases among a much wider range of possibilities. Beyond all-optical data processing, potential applications of coherent metadevices include small signal amplification and coherence filtering^8^.

The switching network demonstrated here is one example among many opportunities arising from fibre-integration of metasurfaces, which could also be used to control, for example, focusing, polarization, spectral characteristics, propagation direction and angular momentum of light^32–34^.

In summary, we report a metamaterial-based fiberized switching network for all-optical signal processing that is compatible with optical telecommunications fibre components. The multi-functional switch can perform effectively-nonlinear signal processing functions including input/output relations analogous to XOR, AND and NOT operations, and the underlying mechanism of coherent transparency and coherent absorption is compatible with single-photon signals and 100 THz bandwidth. We therefore anticipate that such fiberized metadevices may provide solutions for quantum information networks as well as orders-of-magnitude improvements in speed and energy consumption over existing nonlinear approaches to all-optical signal processing in coherent information networks.

## Methods

### Metadevice fabrication

For the realization of the metadevice, the metamaterial was fabricated on the cleaved end face of a polarization-maintaining single-mode fibre as described above. The metamaterial nanostructure was fabricated with its symmetry axis aligned with the slow axis of the Panda-style fibre. In order to allow metamaterial illumination from both sides, the metamaterial-covered fibre was interfaced with a second polarization-maintaining fibre using a microcollimator arrangement consisting of a pair of microlenses. Specifically, the metamaterial-covered fibre end was inserted into a fibre ferrule and positioned at the focal point of an anti-reflection-coated microlens to fabricate a compact microcollimator that was stabilized by a glass capillary shell. The second cleaved fibre tip was mounted in the same way. Using kinematic mounts, the two microcollimators were aligned with each other by maximizing optical coupling efficiency and polarization contrast. The aligned microcollimator arrangement was then fixed with UV-cured adhesive, stabilized with an outer glass capillary shell and placed in an anodized aluminum enclosure for additional protection (Fig. 2a).

### Experimental metadevice characterization

Experimental characterization was performed using the interferometer arrangement presented in Fig. 2a. All fibre components were based on Panda-style polarization-maintaining single-mode fibres. In all cases the incident electric field was oriented parallel to the symmetry axis of the metamaterial nanostructure (slow axis of the fibre). Measurements at kHz frequencies used the 180 μW output of a fibre-coupled 1550 nm CW laser diode and were recorded using InGaAs photodetectors and an oscilloscope (Agilent Technologies DSO7104A). They were calibrated by taking into account the insertion losses of fibre components, such that *I*_α_, *I*_β_, *I*_γ_ and *I*_δ_ in Figs. 2b, c and 3 correspond to the intensities entering and leaving the metadevice’s fibre connectors. The peak input power at the metadevice input connector *I*_α_ = *I*_β_ was 10 μW. The input signal modulators used were low-loss electro-optical 10 Gbit s^−1^ phase and intensity modulators (EOspace) driven by a waveform generator (AM300 by Rohde & Schwarz).

Measurements at GHz frequencies used a fibre-coupled tuneable CW laser (ID Photonics CoBrite-DX4). Here the EDFA-amplified output power was detected by an oscilloscope (Agilent Infiniium DCA-J 86100C) as described in the main text. The peak input power at the metadevice input connector *I*_α_ = *I*_β_ is ~100 μW in the case of Fig. 4a, b, 30 μW in Fig. 4c and 50 μW in Fig. 5. The modulators used were low-loss electro-optical 10 Gbit s^−1^ phase and intensity modulators (EOspace) driven by an arbitrary waveform generator (Tektronix AWG7122C) and a radio frequency amplifier (LA Techniques) in the case of Fig. 4a, b, and a bit pattern generator (SHF 12100 B) in the case of Figs. 2a (eye diagrams), 4c and 5.

### Data availability

The data from this paper is available from the University of Southampton ePrints research repository: 10.5258/SOTON/D0172.

## Electronic supplementary material

Supplementary Information
