# Supplementary material for: Fibre-optic metadevice for all-optical signal modulation based on coherent absorption
Source: Nat Commun. 2018 Jan 12;9:182. doi: 10.1038/s41467-017-02434-y (PMC5766546; doi:10.1038/s41467-017-02434-y)
Supplement: Supplementary file 1 — Supplementary Information [file 41467_2017_2434_MOESM1_ESM.pdf]

Supplementary Note 1: Coherent absorption of femtosecond pulses

While our experimental equipment does not allow us to test the performance of the main manuscript's fiberized modulator beyond 40 Gbit s^{-1}, we expect that the metadevice can in principle operate at much higher frequencies. Indeed, it was recently shown that the underlying phenomena of coherent absorption and coherent transparency in plasmonic metamaterials occur on timescales as short as 10 fs implying a potential bandwidth on the order of 100 THz [1]. We demonstrate this by measuring coherent absorption of femtosecond pulses in a freestanding plasmonic metamaterial that is similar to the one employed in our metadevice, see Supplementary Fig. 1a. The metamaterial consists of a freestanding gold film of 60 nm thickness perforated with a split ring aperture array of a smaller 320 nm period and it exhibits an absorption peak at the experimental wavelength of 800 nm that was determined by the available ultra-short pulse laser. Our measurements show that coherent absorption starts to drop for pulses shorter than 11 fs. We note that a slow decline of coherent absorption upon increase of pulse duration from 11 fs to 185 fs is explainable by the onset of nonlinear absorption at high fluences. The duration of the shortest optical pulse that can be efficiently absorbed is linked to the plasmon relaxation time in gold and the spectral width of the plasmonic response (Supplementary Fig. 1b) as interaction of spectral components of pulses outside of the plasmonic absorption line will not lead to efficient coherent absorption. The absorption resonance of our metadevice has roughly the same spectral width in terms of wavelength, but operates at about twice the wavelength. Assuming the same time bandwidth product in both cases, this implies that our metadevice will efficiently absorb pulses as short as 40 fs, corresponding to a potential bandwidth of tens of THz for our device (Supplementary Fig. 1c). We note, however, that such bandwidth will be difficult to realize in a fiberized device due to dispersion limitations of the fibres.

The metamaterial absorber for experiments with femtosecond pulses (Supplementary Fig. 1a,b) is a nanostructured free-standing gold film of 60 nm thickness. It was fabricated by thermal evaporation of gold on a 50-nm-thick silicon nitride membrane, followed by silicon nitride removal by reactive ion etching and nanostructuring of the remaining free-standing gold film by gallium focused ion beam milling. The gold film is perforated with an array of 320×320 nm^2 split ring apertures that has an overall size of 50×50 µm^2 and a resonant absorption peak around 800 nm wavelength.

Coherent absorption was measured as a function of pulse duration using a free-space setup and a 6 fs mode-locked Ti:sapphire laser (Femtolasers Rainbow) operating at a central wavelength of 800 nm and equipped with a pulse shaper (Biophotonics MIIPS). This was performed by splitting the output of the pulse shaper along two paths of identical length, which are recombined on the metamaterial absorber such that constructive interference occurs on the metamaterial and the light that remained after interaction with the nanostructure was detected.

The absorption spectra of the metamaterial used in the metadevice of the main manuscript (Supplementary Fig. 1c) were modelled by simulating a single metamaterial unit cell with periodic boundary conditions and normal incidence illumination using finite element modelling (COMSOL Multiphysics 3.5a) in three dimensions. The permittivity of gold was taken from Supplementary Reference 2 and the permittivity of glass was assumed to be 2.0736.

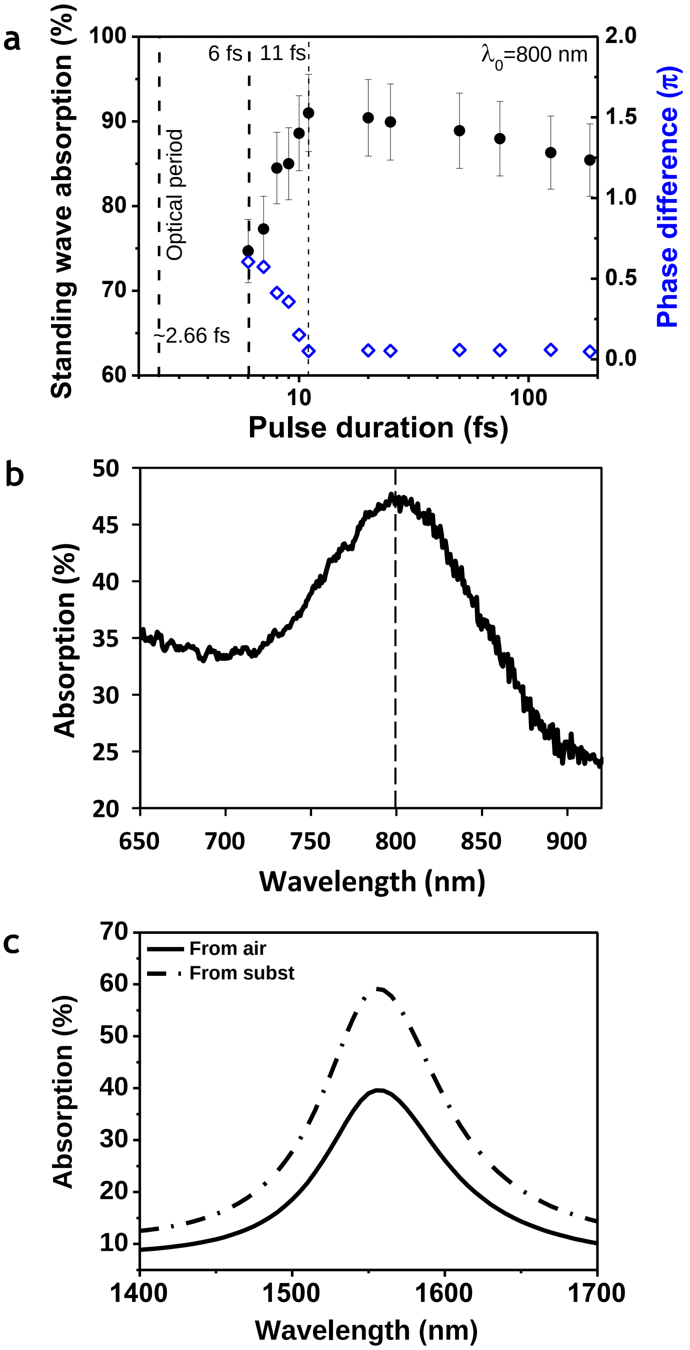

Supplementary Figure 1: **Interaction of femtosecond pulses with a thin absorber.** **a** Coherent absorption of counter-propagating femtosecond pulses measured on a freestanding plasmonic metamaterial for different pulse durations at 800 nm wavelength. The phase difference between the output beams (diamonds) becomes dependent on the pulse duration as absorption (circles) deteriorates for pulses shorter than 11 fs. **b** Measured absorption spectrum of the plasmonic metamaterial used in panel **a** for illumination by a single beam of light. **c** Simulated absorption spectrum of the metamaterial used in the metadevice of the main manuscript for illumination from outside the fibre (solid, α) and within the fibre (dashed, β).

Supplementary References

[1] Nalla, V., Valente, J., Sun, H. & Zheludev, N. I. 11-fs dark pulses generated via coherent absorption in plasmonic metamaterial. *Opt. Express* **25**, 22620–22625 (2017).

[2] Palik, E. D. *Handbook of Optical Constants of Solids* (Academic Press, 1985).
